# A comparative analysis of peptide-delivered antisense antibiotics using diverse nucleotide mimics

**DOI:** 10.1261/rna.079969.124

**Published:** 2024-06

**Authors:** Chandradhish Ghosh, Linda Popella, V. Dhamodharan, Jakob Jung, Julia Dietzsch, Lars Barquist, Claudia Höbartner, Jörg Vogel

**Affiliations:** 1 Helmholtz Institute for RNA-based Infection Research (HIRI), Helmholtz Centre for Infection Research (HZI), D-97080 Würzburg, Germany; 2 Institute of Molecular Infection Biology (IMIB), University of Würzburg, D-97080 Würzburg, Germany; 3 Cluster for Nucleic Acid Therapeutics Munich (CNATM), Munich, Germany; 4 Institute of Organic Chemistry, Center for Nanosystems Chemistry, University of Würzburg, 97074 Würzburg, Germany; 5 Faculty of Medicine, University of Würzburg, 97080, Würzburg, Germany

**Keywords:** antibiotics, antimicrobial resistance, antisense oligomers, nucleic acid mimics, RNA-seq

## Abstract

Antisense oligomer (ASO)-based antibiotics that target mRNAs of essential bacterial genes have great potential for counteracting antimicrobial resistance and for precision microbiome editing. To date, the development of such antisense antibiotics has primarily focused on using phosphorodiamidate morpholino (PMO) and peptide nucleic acid (PNA) backbones, largely ignoring the growing number of chemical modalities that have spurred the success of ASO-based human therapy. Here, we directly compare the activities of seven chemically distinct 10mer ASOs, all designed to target the essential gene *acpP* upon delivery with a KFF-peptide carrier into *Salmonella.* Our systematic analysis of PNA, PMO, phosphorothioate (PTO)-modified DNA, 2′-methylated RNA (RNA-OMe), 2′-methoxyethylated RNA (RNA-MOE), 2′-fluorinated RNA (RNA-F), and 2′–4′-locked RNA (LNA) is based on a variety of in vitro and in vivo methods to evaluate ASO uptake, target pairing and inhibition of bacterial growth. Our data show that only PNA and PMO are efficiently delivered by the KFF peptide into *Salmonella* to inhibit bacterial growth. Nevertheless, the strong target binding affinity and in vitro translational repression activity of LNA and RNA-MOE make them promising modalities for antisense antibiotics that will require the identification of an effective carrier.

## INTRODUCTION

Microbial resistance to frontline drugs has necessitated the search for alternatives to conventional antibiotics. Short single-stranded antisense oligomers (ASOs) that inhibit the translation of essential bacterial genes are promising candidates (Ghosh et al. 2019; Vogel 2020). They have been shown to be effective antibacterials against a range of pathogens (Geller et al. 2003, 2005; Ghosal and Nielsen 2012; Soofi and Seleem 2012; Martínez-Guitián et al. 2020; da Silva et al. 2021) and their efficacy has also been validated in animal models of infection with *Escherichia coli*, *Pseudomonas aeruginosa*, and *Klebsiella pneumoniae* either as monotherapy or in combinations with other antibiotics (Sully et al. 2017; Geller et al. 2018; Sturge et al. 2019; Moustafa et al. 2021).

Antisense antibiotics have advantages over conventional antibiotics. First, given their sequence-specific mode of action, they can be designed to be species-specific, thus allowing selective targeting of pathogenic bacteria over commensals (Mondhe et al. 2014; Ayhan et al. 2016; Vogel 2020). Second, the development of resistance due to mutations in the target mRNA can be reversed by changing the ASO sequence (Eller et al. 2021). Third, ASOs can in principle target any transcript of interest and can, therefore, also be used to reinstate the sensitivity of drug-resistant bacteria to existing antibiotics (Daly et al. 2017; Sturge et al. 2019). Moreover, antisense technology can enable the manipulation of particular bacterial species in a microbial community. Nevertheless, despite the recent successes of ASO-based therapeutics to treat human diseases (Egli and Manoharan 2023), no antisense antibiotic has yet been approved for clinical use, and improvements in their design and delivery are needed. Unfortunately, due to the inherent differences between eukaryotic and bacterial cells, the design principles underlying ASO activity and uptake cannot be easily transferred.

Because bacteria lack eukaryotic uptake mechanisms, such as endocytosis, phagocytosis, or (macro-)pinocytosis, alternative routes need to be used for intrabacterial ASO delivery. Therefore, antibacterial ASOs are generally modular, comprising the antisense effector and a delivery vehicle, such as cell-penetrating peptides (CPPs). The challenge to deliver ASOs across the complex bacterial envelope also restricts their length. Although bacterial ASOs typically span 10–15 nt, their eukaryotic counterparts tend to be longer than 20 nt, resulting in substantial differences in their hybridization strengths. Bacterial ASOs are designed to hybridize to the ribosome-binding site (RBS) or start codon of an mRNA of interest, thereby sterically blocking ribosome binding and preventing translation of the target protein (Shen and Corey 2018). Eukaryotic ASOs can act through additional mechanisms, including RNase H1-mediated cleavage and degradation of target transcripts, AGO2-mediated degradation, modulation of splicing or polyadenylation, nonsense-mediated mRNA decay (NMD), and also up-regulation of gene expression (Crooke et al. 2021), which influences their design. Therefore, the lessons learned in enhancing ASO therapy for human diseases cannot be directly applied to the design of antibacterial ASOs. However, given the amount of pharmacological and clinical data obtained with approved ASO drugs, a systematic assessment of those modalities for potential application in bacteria seems warranted.

Due to the rapid degradation of unmodified RNA by nucleases, ASOs are based on modified nucleic acids or mimetics with improved stability and nuclease resistance. To date, peptide nucleic acid (PNA) and phosphorodiamidate morpholino (PMO) have been the most popular modalities for the use as antisense antibiotics (Iubatti et al. 2022). In PNAs, the phosphodiester backbone of the nucleic acid is replaced by a pseudo-peptide backbone, whereas in PMOs the five-membered ribose sugar is replaced by a six-membered morpholine ring. In contrast to oligonucleotide-based ASOs, which are negatively charged, both PNA and PMO are neutral in charge. This improves their target binding affinity and eases delivery by cationic CPPs across the negatively charged bacterial membrane.

**FIGURE 1.**
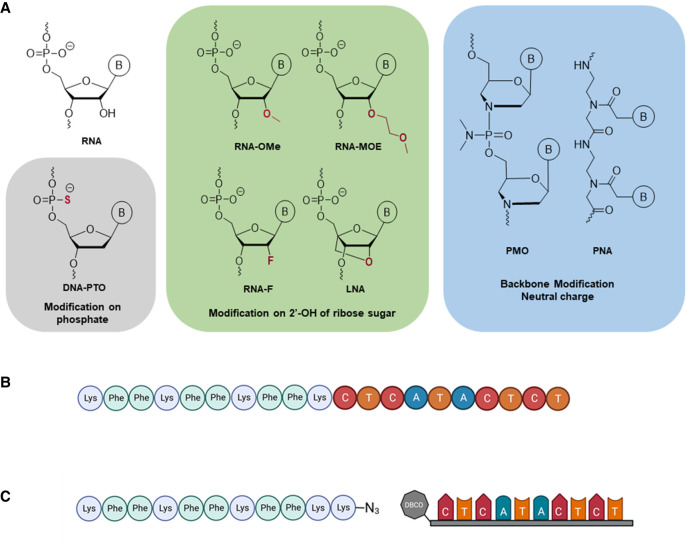
Design and synthesis of the different ASOs used in the study. (*A*) The different chemical modalities used in this study can be grouped into three different categories: modifications of the phosphate backbone (gray), modifications of the ribose sugar (green), and alternative backbone chemistries (blue). The gray and green boxes comprise ASOs that bear net negative charges; ASOs in the blue box are charge-neutral. (*B*) The KFF-PNA conjugates were synthesized as fusion peptides on a peptide synthesizer. (*C*) Schematic for the strain-promoted alkyne–azide click reaction between the peptide (synthesized on a peptide synthesizer) and the ASO (synthesized on an oligonucleotide synthesizer). Created with BioRender.com.

Efforts in medicinal chemistry have led to a large range of additional modifications that enable ASO optimization in terms of pharmacokinetics, pharmacodynamics, and bioavailability. These have been applied to oligonucleotide drug design in eukaryotic systems and several oligonucleotides have been approved for clinical use against a range of human diseases such as retinitis, macular degeneration, hypercholesterolemia, amyloidosis, and muscular dystrophy (Crooke et al. 2021). Successful ASO modalities include modifications on the 2′-hydroxy group on the ribose sugar of a nucleotide, such as 2′–4′ carbon bridged nucleic acids (BNA) and locked nucleic acids (LNA), 2′-methylated RNA (RNA-OMe), 2′-*O*-methoxyethyl (2′-MOE), and 2′-Fluoro (2′-F). Another common modification is the substitution of the nonbridging oxygen in the phosphate backbone of nucleic acids, for example, phosphorothioate (PTO)-modified DNA (Fig. 1A; Harth et al. 2000; Meng et al. 2009, 2015; Lopez et al. 2015; Chen et al. 2016b; Hegarty et al. 2016). Few studies thus far have explored the antibacterial potential of these ASO modalities. 2′-MOE and 2′-F have not been tested in bacteria at all. Even in cases where different ASOs have been tested with the same delivery methods, conflicting results have been observed for neutral and negatively charged ASOs (negASOs) (Równicki et al. 2017; Pereira et al. 2021). Moreover, there has been no side-by-side comparison of the efficacy of these different ASO chemistries in terms of target binding, inhibition of target gene translation, and their antibacterial activity.

Over the years, methodology has been developed to study the uptake, target binding, and specificity of ASO antibiotics. For example, the Nielsen lab has established methods to study the internalization of peptide-PNA conjugates in bacteria (Yavari et al. 2021). Our own efforts have been directed at using RNA sequencing (RNA-seq) to read out how ASOs affect target mRNA levels in bacteria and how bacteria respond to ASO treatment in general (Popella et al. 2021, 2022). This is complemented by the development of assays and algorithms to study ASO potency and specificity in vitro and in vivo (Hör et al. 2022; Popella et al. 2022; Jung et al. 2023). Here, using these tools, we have systematically compared seven ASO modalities in their antibacterial efficacy. We analyzed their binding affinity and inhibition of target protein synthesis in a cell-free system and assessed their effect on the growth and physiology of the model pathogen *Salmonella enterica* (henceforth, *Salmonella*) upon conjugation to the commonly used carrier peptide (KFF)_3_K (henceforth, KFF). Additionally, we studied the delivery of these ASOs across *Salmonella* membranes. Our results suggest that although LNA and 2′-methoxyethylated RNA (RNA-MOE) tightly bind to and efficiently inhibit translation of their target mRNA in vitro, the KFF carrier peptide is unable to deliver these negASOs into *Salmonella*. This reinforces the use of neutral backbone ASOs for KFF-mediated delivery, but also indicates that negASOs might be efficient antisense antibacterials when delivered with a compatible carrier. Moreover, we provide a direct comparison between the impacts of PNA and PMO treatments, examining both the down-regulation of target transcripts (via RNA-seq) and the reduction of target protein levels. While performed in *Salmonella*, this study establishes a general experimental framework for the systematic exploration of alternative ASO modalities in other microbes.

## RESULTS

### Study design

For our side-by-side comparison of different ASO modalities, we selected three representatives of backbone-modified synthetic nucleotides (PNA, PMO, and PTO) and four ribose-modified nucleotides (2′-OMe, 2′-MOE, 2′-F, and LNA); members of both groups are successfully used as ASO therapeutics for human diseases (Crooke et al. 2021). PNA and PMO are charge-neutral, whereas all the other ASOs mentioned above are negatively charged due to their phosphate backbone (Fig. 1A; Supplemental Fig. S1; Table 1). We chose to target the essential gene *acpP* (acyl carrier protein), because PNA-mediated silencing of AcpP protein synthesis rapidly and effectively inhibits *Salmonella* growth (Popella et al. 2021). In addition, the concomitant and selective depletion of *acpP* mRNA by an as-yet-unknown mechanism can be read out by RT-qPCR, northern blot, or RNA-seq (Popella et al. 2021). Each of the seven different ASOs was designed to be complementary to nucleotides −5 to +5 relative to the translational start site of *acpP* (sequence of the ASOs: CTCATACTCT). We considered a 10mer ASO optimal to balance hybridization strength and efficient cellular uptake, because increasing ASO length hinders bacterial internalization (Hegarty and Stewart 2018; Goltermann et al. 2019).

**TABLE 1.** The different chemical modalities used in this study and their physical parameters

Chemical entity	Net charge	Molecular mass	*T*_m_ of ASO:mRNA complexes (°C)	mRNA-binding affinity (EC_50_, nM)^a^
RNA-F	−10	3540.55	56.5 ± 0.9	72.8 ± 36.2
RNA-OMe	−10	3660.75	48.3 ± 0.8	4.0 ± 0.2
RNA-MOE	−10	4101.02	55.6 ± 0.6	0.3 ± 0.2
LNA	−10	3752.72	87.4 ± 2	0.3 ± 0.1
DNA-PTO	−10	3576.48	25.8 ± 0.7	Could not be determined
PMO	0	3936.5	41.8 ± 0.6	5.9 ± 2.7
PNA	0	2579.02	44.3 ± 1.1	0.4 ± 0.4
(KFF)_3_KK(N_3_)	+5	1565.90	NA	NA
(KFF)_3_K	+5	1427.79	NA	NA

(RNA-F) 2′-fluorinated RNA, (RNA-OMe) 2′-methylated RNA, (RNA-MOE) 2′-methoxyethylated RNA, (LNA) 2′–4′-locked RNA, (PTO) phosphorothioate, (PMO) phosphorodiamidate morpholino, (PNA) peptide nucleic acid, (NA) not applicable.

^a^Mean ± standard deviation (two independent experiments).

For experiments involving delivery into *Salmonella*, we fused the different ASOs to the KFF carrier peptide (Fig. 1B,C), a commonly used CPP. Although the covalent conjugation of the cationic amphiphilic KFF peptide and the anionic ASOs might lead to the formation of aggregates due to electrostatic interactions, we chose KFF for three reasons. First, KFF has previously been used as carrier for different ASO modalities (such as PNA, PMO, and LNA) and has been successfully applied to different bacterial species (Alajlouni and Seleem 2013; Meng et al. 2015; Martínez-Guitián et al. 2020; da Silva et al. 2021). For example, a KFF-delivered 21-mer LNA-based ASO targeting *S. aureus* has been shown to be active both in vitro and in a murine model of infection (Meng et al. 2015). Second, in a mini-library of different CPP-ASOs, KFF-delivered ASOs had the smallest global effect on the bacterial transcriptome (Popella et al. 2021). Third, it was recently shown that upon translocation into bacteria, the peptide is cleaved within the periplasm leaving the ASO or truncated peptide–ASO conjugates for further uptake into the cytoplasm (Yavari et al. 2021). This minimizes the effects of the carrier peptide on target binding.

The conjugates were produced by two different methods: PNA by whole-peptide synthesis (Fig. 1B) and all other ASOs by copper-free click chemistry (see Materials and Methods; Fig. 1C; Supplemental Fig. S2). Consequently, we included two different delivery peptides as controls in the experiments described below: (KFF)_3_K for PNA and (KFF)_3_KK(N_3_) for all other ASOs.

### RNA-MOE, LNA, and PNA have comparable binding affinity to the target mRNA in vitro

**FIGURE 2.**
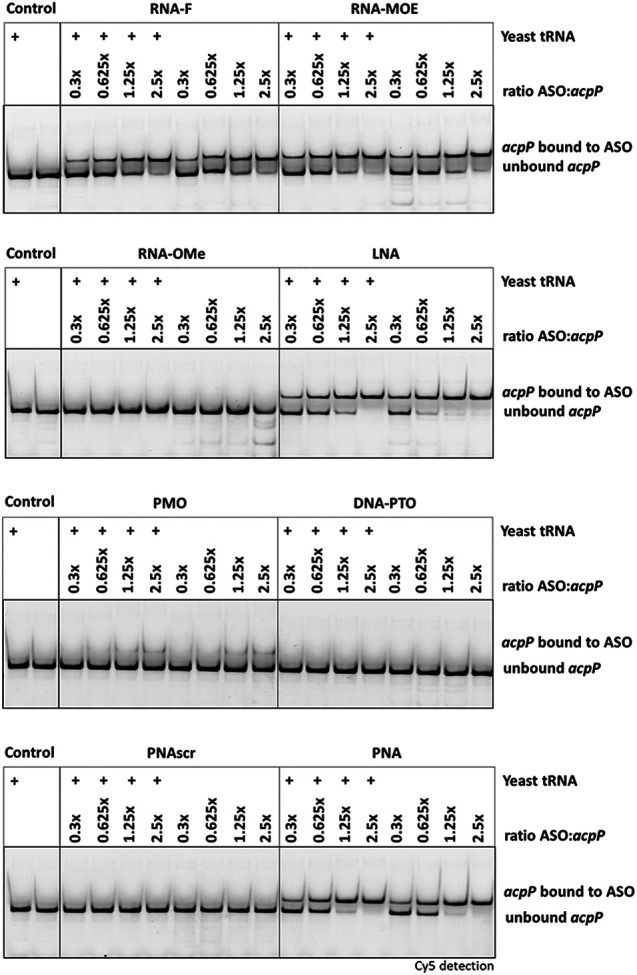
Electrophoretic mobility shift assay. Retardation of electrophoretic mobility of a 5′ Cy5-labeled *acpP* target RNA (40 nt) dependent on ASO hybridization. Different ASO modalities were tested at 2.5:1, 1.25:1, 0.6:1, and 0.3:1 molar ratios of ASO:RNA as indicated. Yeast tRNA (10 µg) was spiked in samples labeled with “+.” Samples were separated on 15% native PAA gels, and one representative example of two independent experiments is shown.

To compare the ability of the unconjugated ASOs to hybridize to the target transcript, we used electrophoretic mobility shift assays (EMSAs), thermal UV melting curves, and microscale thermophoresis (MST). We first conducted EMSAs using a 40 nt target RNA sequence (nt −20 to +20 relative to the *acpP* translational start codon), which was 5′-labeled with a Cy5 dye. Sequence-specific ASOs were added at defined ASO:RNA ratios, while water or a scrambled PNA (PNAscr) were used as negative controls. We performed these binding studies with unconjugated ASOs, because the KFF carrier peptide is cleaved during transport into the bacterial cytoplasm (Yavari et al. 2021). For RNA-F, RNA-MOE, LNA, and PNA, we observed a concentration-dependent shift of the target mRNA, starting at a 0.3:1 ratio of ASO:RNA (Fig. 2). Surprisingly, PMO showed limited binding even at 2.5× excess. RNA-OMe, DNA-PTO, and the PNAscr control did not interact with the target mRNA at any of the concentrations tested. The DNA-PTO modification is known to reduce the binding affinity of the ASO to its target RNA (Chan et al. 2006), and a 10mer might be too short to mediate interactions under our experimental conditions. To test if an excess of unspecific RNA impacts target binding, we also included samples that contained 1 μg yeast tRNA. We found that the hybridization to the target was largely unaffected by the presence of yeast tRNA. In the case of LNA, a small decrease in binding was seen for the ratios of 0.625× and 1.25×.

**FIGURE 3.**
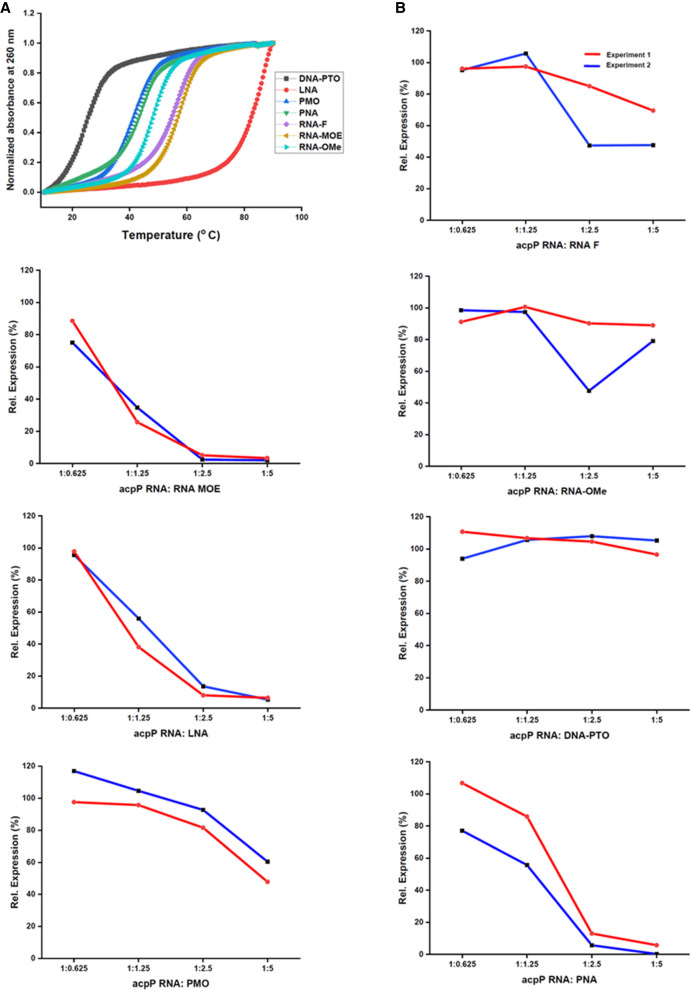
ASO:RNA duplex binding studies and in vitro translation assays. (*A*) Melting curves of *acpP* RNA–ASO complexes at equimolar concentrations. The target RNA used in this experiment is 10 nt long. (*B*) The capacity of the ASOs to inhibit translation of an *acpP::gfp* reporter transcript in vitro was analyzed at 5:1, 2.5:1, 1.25:1, and 0.6:1 molar ratios of ASO:RNA. Graphs show Image J quantitation of the GFP fusion protein in a western blot analysis of two independent experiments (replicates are color-coded in red and blue). Protein expression levels are shown relative to the water control. The corresponding western blots are shown in Supplemental Figure S5.

Next, we assessed the melting temperatures of each ASO:RNA duplex across a temperature range of 10°C–90°C (Fig. 3A; Supplemental Fig. S3). In this experiment, we used a fully complementary 10mer RNA fragment instead of the 40 nt long target RNA sequence, because the longer target might form internal secondary structures that could interfere with the measurement of the melting temperature. After normalizing the melting curves of all the ASO:RNA complexes for direct comparison, DNA-PTO exhibited the lowest melting temperature (25.8°C) while LNA exhibited the highest melting temperature (87.4°C) (Fig. 3A; Table 1). This is in line with previous observations (Freier and Altmann 1997) and our EMSA results (Fig. 2). PMO exhibited a *T*_m_ value of 41.8°C, while PNA, in line with previous findings from the Nielsen lab, exhibited a *T*_m_ value of 44.3°C (Goltermann et al. 2019). In contrast, RNA-OMe, which showed little interaction with the target RNA in EMSA studies, demonstrated a higher *T*_m_ value of 48.3°C (Table 1). The melting temperature of RNA-F and RNA-MOE was around 56°C (Table 1).

To further quantify the binding affinities of the ASOs to the target mRNA, we used MST, a sensitive technique that measures a temperature-induced change in the fluorescence of a substrate as a function of the concentration of a nonfluorescent ligand (Moon et al. 2018; Zimmer et al. 2021). MST allowed us to quantify target affinity by calculating EC_50_ values, the half maximal effective concentration of binding to target mRNA (Table 1; Supplemental Fig. S4). RNA-MOE, LNA, and PNA showed the highest target affinity with an EC_50_ in the upper picomolar range. In contrast, the binding affinities of RNA-OMe, PMO, and RNA-F were 10–100 times lower, with EC_50_ values in the lower-mid nanomolar range (Table 1). These EC_50_ values for RNA-F, RNA-OMe, and PMO do not fully reflect the EMSA binding pattern, possibly due to technical differences between the methods. Nevertheless, we can conclude that RNA-MOE and LNA bound to the target mRNA at affinities comparable to the neutral PNA. Both are known to adopt C3′-endo ribose pucker conformation, which enhances their binding affinity to target mRNA (Julien et al. 2008; Pallan et al. 2012).

### RNA-MOE, LNA, and PNA are strong inhibitors of translation in a cell-free system

As a next step, we used a cell-free in vitro translation system (Popella et al. 2022) to investigate whether ASO binding to mRNA leads to a block in protein synthesis. As a template, we used the *acpP* sequence spanning −40 to +51 relative to the AUG start codon fused to a *gfp* reporter gene (*acpP*::*gfp*). This construct was in vitro transcribed, preincubated with different ASOs at different ratios and subsequently translated in vitro. Water was used as a negative control. Quantifying the translated AcpP(1–17)::GFP fusion protein by western blot analysis, we observed that the high-affinity ASOs RNA-MOE, LNA, and PNA were effective inhibitors, substantially blocking in vitro translation of *acpP*::*gfp* at 1.25× molar excess (Fig. 3B; Supplemental Fig. S5). Inhibition of in vitro translation was less pronounced in the presence of RNA-F, PMO, or RNA-OMe. DNA-PTO, which is unable to bind to the target mRNA under our experimental conditions, did not inhibit protein synthesis (Fig. 3B; Supplemental Fig. S5).

### Only PNA and PMO show antibacterial activity

**FIGURE 4.**
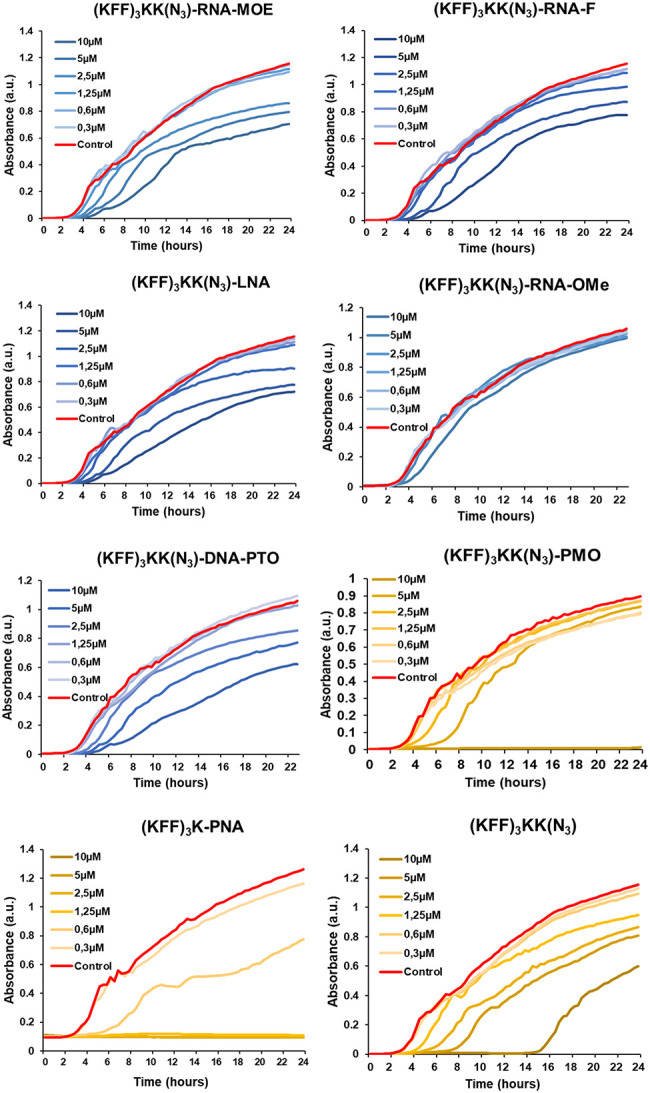
Antibacterial activity of KFF-ASO conjugates. Growth of *Salmonella* was monitored in the absence or presence of varying concentrations of seven different KFF-ASO conjugates or the (KFF)_3_KK(N_3_) peptide control. PNA, PMO, and the cationic carrier peptide control are depicted in shades of yellow, whereas negASOs are in shades of blue. Water control is shown in red. Graphs are representative examples of two independent experiments.

Next, we sought to compare the antibacterial efficacy of the ASOs upon delivery into bacterial cells with a KFF carrier peptide. Therefore, we measured the growth kinetics of *Salmonella* in the presence of the ASOs for 24 h (Fig. 4). Because we have previously shown that the KFF peptide has a minimum inhibitory concentration (MIC) of >10 µM (Popella et al. 2021), we choose 10 µM as a cutoff point in our experiments. As expected, none of the unconjugated ASOs showed any antibacterial activity against *Salmonella* (Supplemental Fig. S6A), reiterating the importance of the carrier peptide. When delivered by the KFF peptide, PNA inhibited the growth of *Salmonella* at 1.25 µM (as reported before [Popella et al. 2021]) and PMO did so at 10 µM (Fig. 4). However, none of the negASOs completely inhibited bacterial growth at the concentrations tested, although a slight growth retardation was observed at 5 and 10 µM for RNA-MOE, LNA, RNA-F, and DNA-PTO conjugates. This is likely due to contributions of the KFF peptide, because the unconjugated peptide control showed the same effect. The RNA-OMe conjugate lacked any antibacterial activity. Spotting of the ASO-treated bacterial samples at 24 h confirmed that PMO and PNA were bactericidal at 10 and 1.25 µM, respectively (Supplemental Fig. S6B). In conclusion, despite their high binding affinities for target mRNA in cell-free systems, RNA-MOE and LNA do not effectively inhibit bacterial growth, whereas PNA and PMO do.

### Global transcriptomic changes upon treatment of *Salmonella* with different ASO modalities

**FIGURE 5.**
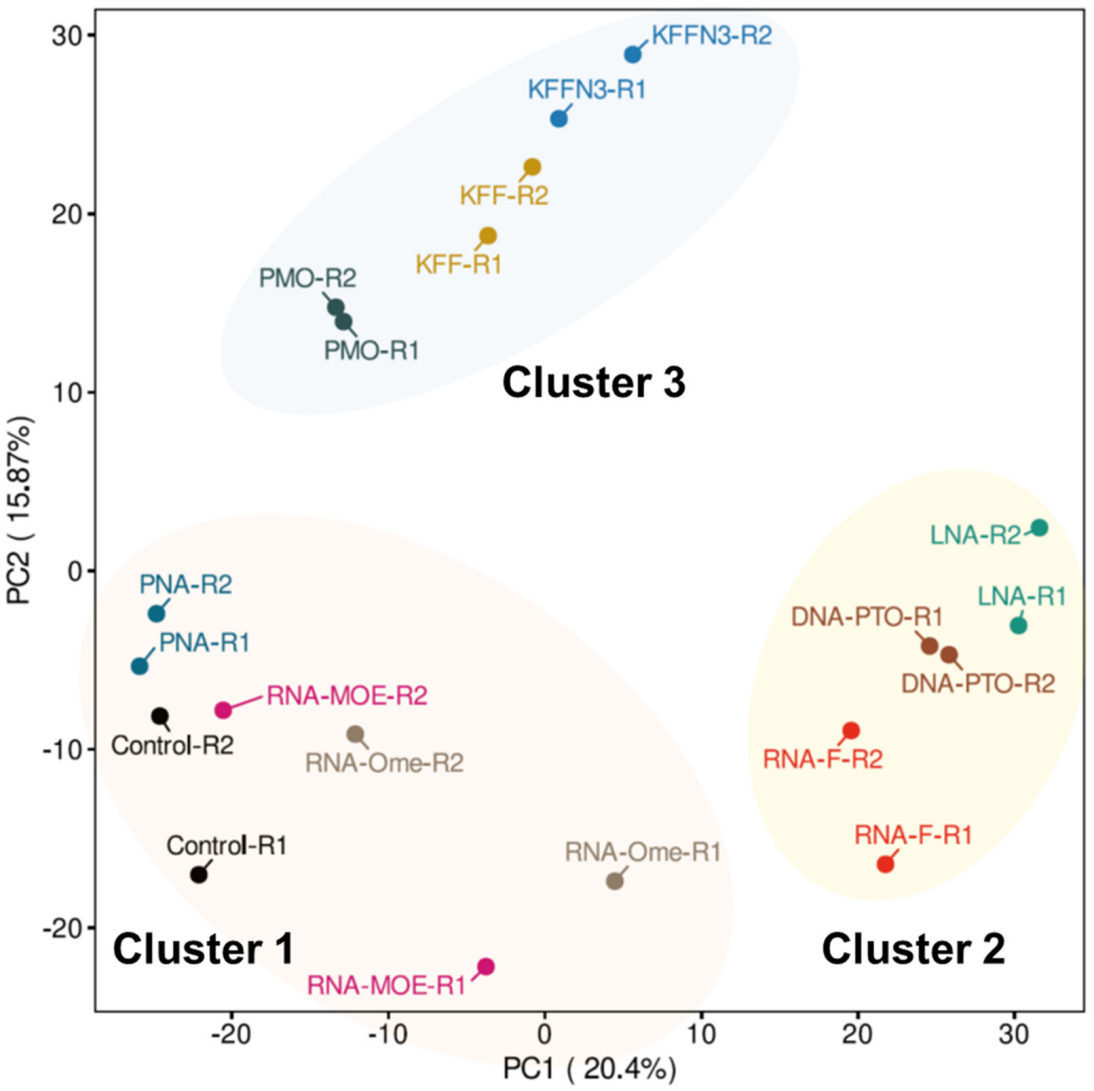
Global transcriptomic signatures of KFF-ASO-treated *Salmonella*. PCA of two independent RNA-seq experiments. The PCA plot shows a projection of the RNA-seq data onto the first two principal components. Treatment conditions separate into three clusters, as shown by the manual addition of cluster-ellipses in the PCA plot.

**FIGURE 6.**
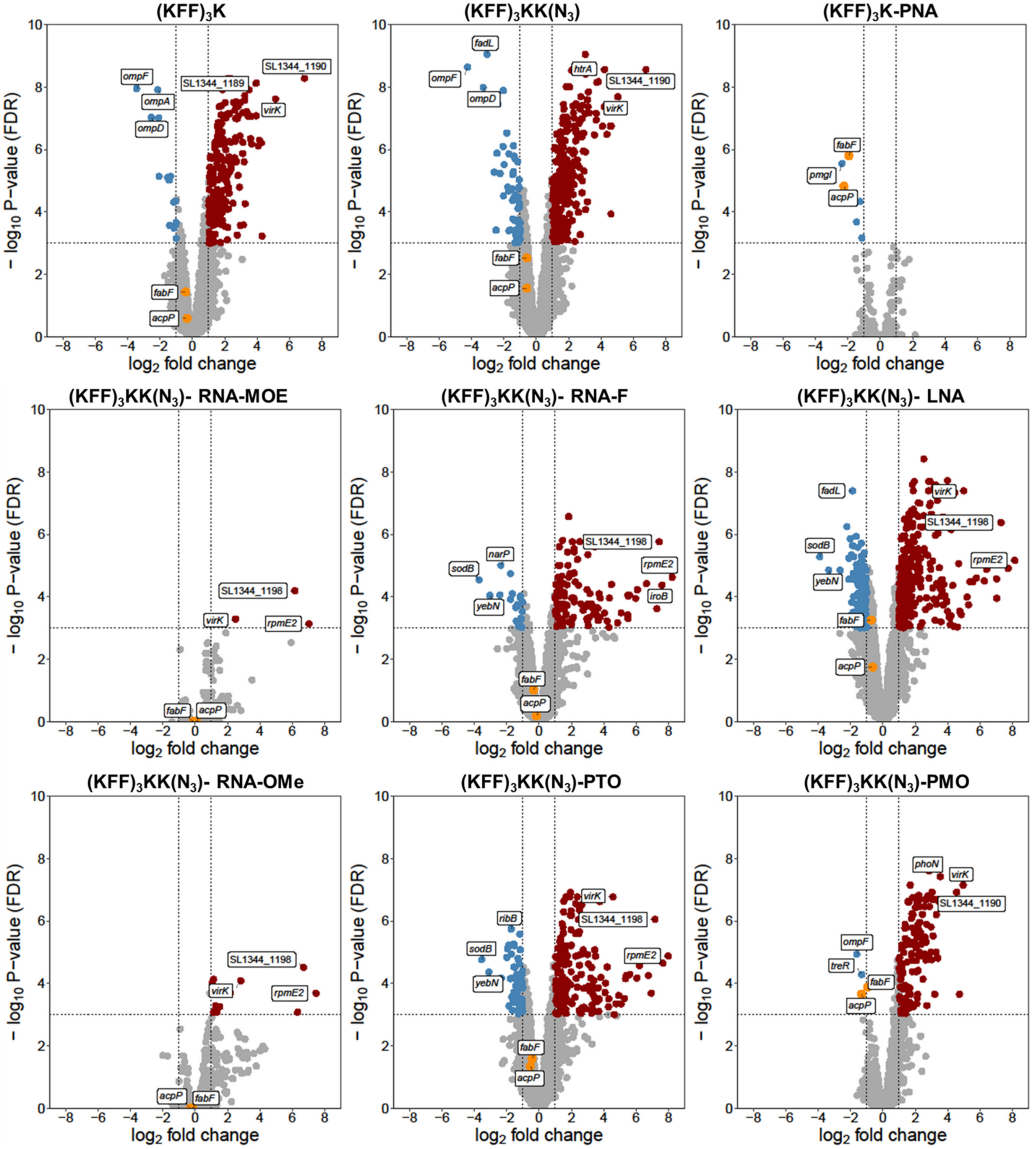
Transcriptomic responses of *Salmonella* upon KFF-ASO treatment. Volcano plots show calculated changes in *Salmonella* gene expression as FDR-adjusted *P*-value (−log_10_, *y*-axis) and fold change (FC) (log_2_, *x*-axis). Significantly differentially regulated genes are characterized by an absolute FC > 2 (down-regulated log_2_ < −1, up-regulated log_2_ > 1; vertical dashed line) and an FDR-adjusted *P*-value < 0.001 (−log_10_ > 3, horizontal dashed line). Significantly down-regulated genes are highlighted in blue, up-regulated genes are highlighted in red. The top three up- or down-regulated transcripts are labeled.

**FIGURE 7.**
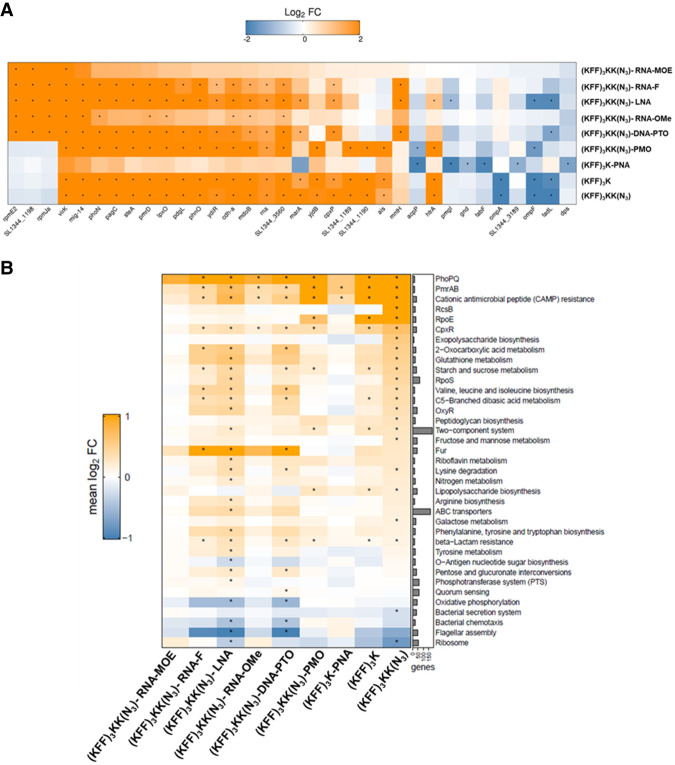
Analysis of differentially expressed genes, KEGG pathways, and regulons. (*A*) Heatmap showing the most strongly differentially expressed genes for all KFF-ASO conjugates. Duplicate RNA-seq samples of *Salmonella* treated for 15 min with KFF-ASOs were normalized to untreated control samples. Log_2_FCs are indicated by color. Orange and blue colors indicate up- and down-regulation, respectively. The heatmap includes the top 10 regulated transcripts per condition. Asterisks (*) denote significantly regulated (absolute log_2_FC > 1 and FDR-adjusted *P*-value < 0.001) genes in the respective condition. (*B*) The heatmap shows the mean log_2_FC for genes assigned to the gene sets. Only the 10 most significantly enriched/depleted gene sets per condition are visualized. Asterisks (*) denote statistical significance (FDR-adjusted *P*-value < 0.01) in the respective condition. The bar chart on the *right* shows the number of genes assigned to the respective gene set.

To assay bacterial responses to the different ASO modalities and to determine target mRNA depletion in cellulo, we performed RNA-seq analysis of *Salmonella* challenged with 5 µM KFF-ASO conjugates for 15 min. We first evaluated two individual RNA-seq experiments by principal component analysis (PCA) and observed three distinct clusters (Fig. 5). The PCA cluster 1 grouped the water control, RNA-MOE, RNA-OMe, and the PNA-treated conditions. The treatments in this cluster lead to only small changes in gene expression as compared to the negative control (Fig. 6), confirming our previous observation that KFF coupled to PNA has little effect on the global transcriptome (Popella et al. 2021), and indicating that RNA-MOE and RNA-OMe do not trigger strong transcriptomic responses either. Cluster 2 grouped the other negASOs. As we have previously observed for KFF peptide exposure (Popella et al. 2021, 2022), these treatments led to the activation of a cationic antimicrobial peptide (CAMP) response, including induction of the PhoP/Q and PmrA/B regulons involved in remodeling LPS, and the CpxR regulon that responds to envelope stress (Fig. 7). This indicates that these compounds affect the cell membrane. These treatments also led to the down-regulation of genes responsible for the destruction of superoxide anion radicals (*sodB*) and those involved in manganese efflux (*yebN*) (Figs. 6 and 7). Finally, cluster 3 grouped samples of the unconjugated peptides and the PMO treatment. Similarly to cluster 2, these activated a strong CAMP response (Fig. 7). The observation of differential activation of membrane stress responses of the different ASO conjugates indicates that the cellular response is dependent on both the carrier peptide and the ASO modality and suggests the need for joint optimization of both components in designing ASOs for targeted gene silencing.

**FIGURE 8.**
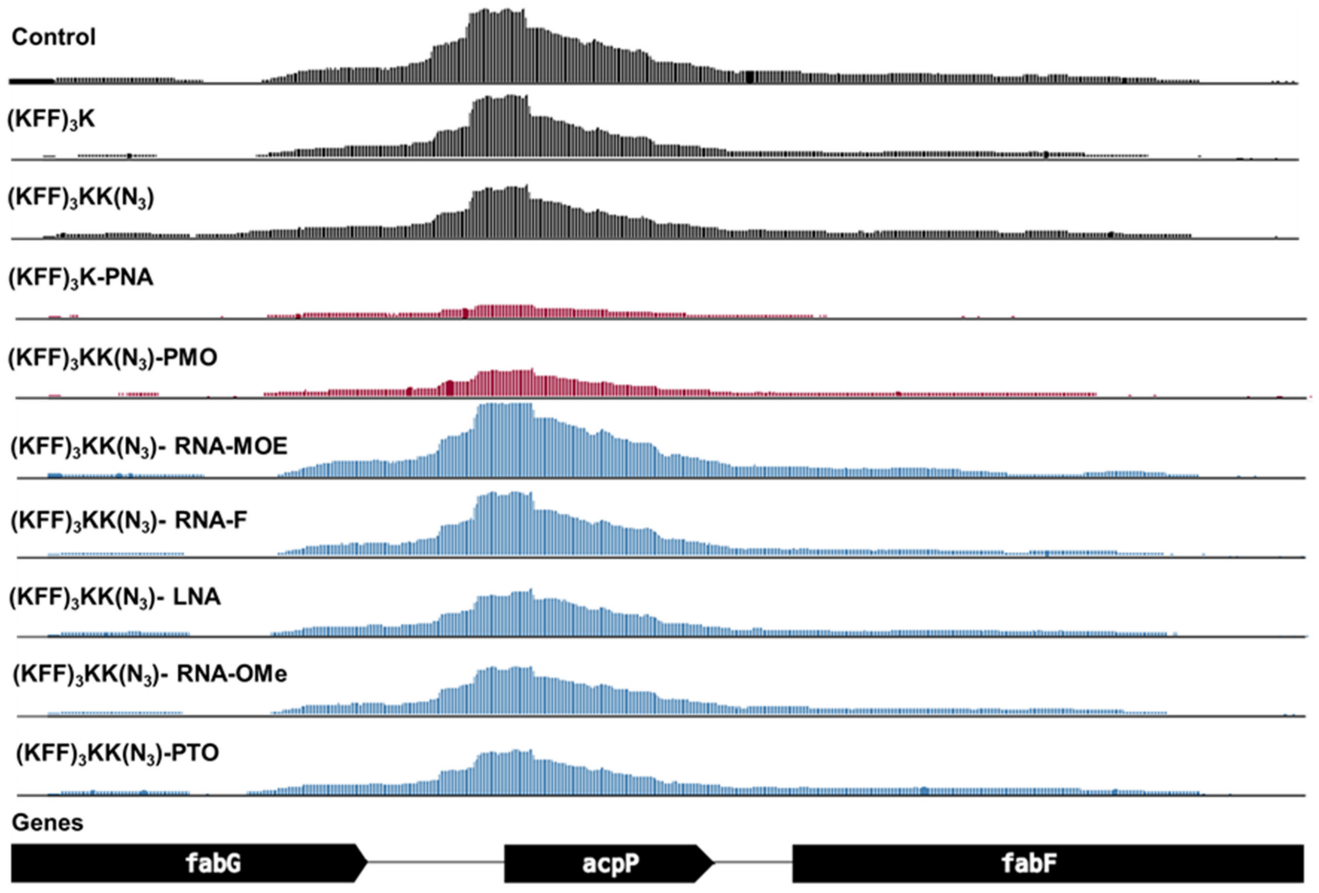
Coverage plot of the acpP transcript and neighboring genes for all tested RNA-seq conditions. The coverage plot shows the abundance of mapped reads normalized by counts per million (CPM). The *y*-axis of all tracks shows the normalized read depth per position, ranging from 0 to 1500 CPM. Control samples without ASO, ASO modalities triggering significant (log_2_FC < −1 and FDR < 0.001) *acpP* depletion and other ASO modalities are colored in black, red, and blue, respectively. One replicate (R1) of the RNA-seq data is shown.

Next, we evaluated target mRNA depletion. In agreement with our previous work (Popella et al. 2021), we found that PNA treatment reduces the levels of *acpP* mRNA (Figs. 6–8). This is also true for PMO treatment (Figs. 6–8). Although the mechanism underlying this phenomenon is still unclear, it might be that reduced ribosome occupancy induced by ASO treatment may increase the sensitivity of the target mRNA to nucleases. Reduction of *fabF* (3-oxoacyl-[acyl carrier protein] synthase 2), a gene that is cotranscribed with *acpP*, was also observed for PNA and PMO (Fig. 6); we have seen this effect before for PNA (Popella et al. 2021). In contrast, none of the negASOs caused significant depletion of the target transcript (false discovery rate [FDR] adjusted *P*-value < 0.01) (Figs. 6 and 8).

In addition to *acpP* and *fabF*, three other genes that directly affect bacterial metabolism were significantly down-regulated by PNA. These genes encoded for DNA binding protein from starved cells (*dps*) and for proteins responsible for decarboxylation of 6-phosphogluconate (*gnd*) and for the interconversion of 2-phosphoglycerate and 3-phosphoglycerate (*pmgI*) (Pardo-Esté et al. 2018). The depletion of *pmgI* by the *acpP*-PNA is due to an off-target binding site in the translation initiation region of *pmgI*, as described before (Hör et al. 2022; Jung et al. 2023). PMO treatment led to the depletion of the transcript encoding the outer membrane (OM) porin OmpF (*ompF*). This might be due to the effects of the KFF peptide, as both sets of carrier peptides also down-regulated transcripts encoding OM porins (*ompA* and *ompF*). Moreover, the pattern of significantly up-regulated transcripts in PMO-treated samples closely resembles that of KFF alone, highlighting the KFF-dependent effects on the transcriptome (Fig. 7A,B).

Overall, we conclude that only ASOs that showed antibacterial activity against *Salmonella* induce target mRNA depletion in vivo within 15 min of treatment and that the overall transcriptomic response differs greatly between different ASO conjugates.

### Treatment with PNA and PMO leads to lower AcpP protein expression in *Salmonella*

**FIGURE 9.**
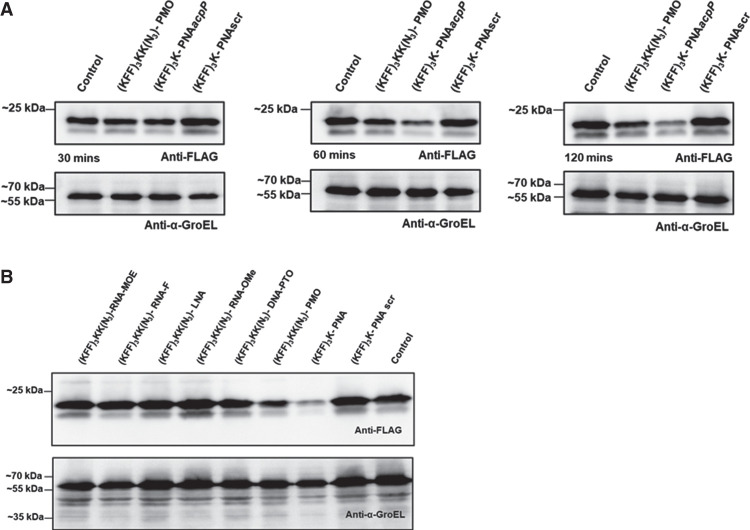
Western blot analysis of AcpP levels upon KFF-ASO treatment of *Salmonella*. (*A*) Western blot analysis of *Salmonella* strain SL1344 expressing AcpP::3xFLAG treated with KFF-PNA or KFF-PMO, each at 5 µM, for 30, 60, and 120 min. (*B*) Western blot analysis of *Salmonella* treated with all *acpP*-targeting KFF-ASO constructs at 5 µM for 120 min. (*A*,*B*) A scrambled KFF-PNA conjugate (PNA scr) and mock-treated (water) samples served as negative controls. Protein lysates were separated on 12% SDS-PAA gels, and proteins were blotted onto nitrocellulose membranes. Membranes were probed with a FLAG-specific antibody to detect the AcpP::3xFLAG fusion protein. GroEL was used as a loading control. The experiments were performed two times and exemplary images are shown.

We have previously shown that PNAs targeting *acpP* lead to rapid depletion of the target transcript (Hör et al. 2022; Popella et al. 2022; Jung et al. 2023), although this is not a universal phenomenon and varies from transcript to transcript (Popella et al. 2022). In other words, an ASO might block target protein synthesis without substantially impacting mRNA levels. Therefore, we wanted to test if the different ASO modalities affect AcpP protein levels in cellulo despite their inability to deplete the *acpP* transcript. We took advantage of *Salmonella* expressing a FLAG-tagged AcpP (AcpP::3xFLAG) protein from its native locus. First, we exposed the bacterial cells to PMO and PNA for 30, 60, or 120 min (Fig. 9A) to determine the best time point for our analysis. PNA-mediated depletion in AcpP levels can be observed from 60 min post-treatment onward, and the effect increases at 120 min post-treatment (Fig. 9A). Similarly, treatment with PMO led to the gradual depletion of AcpP, albeit less pronounced than PNA. We repeated the experiment for all ASOs at the 120 min time point, but we did not observe a detectable reduction in AcpP protein levels upon negASO treatment (Fig. 9B), in line with their inefficacy in inhibiting bacterial growth.

### KFF-mediated permeabilization of the bacterial outer membrane is compromised by conjugation with negASOs

**FIGURE 10.**
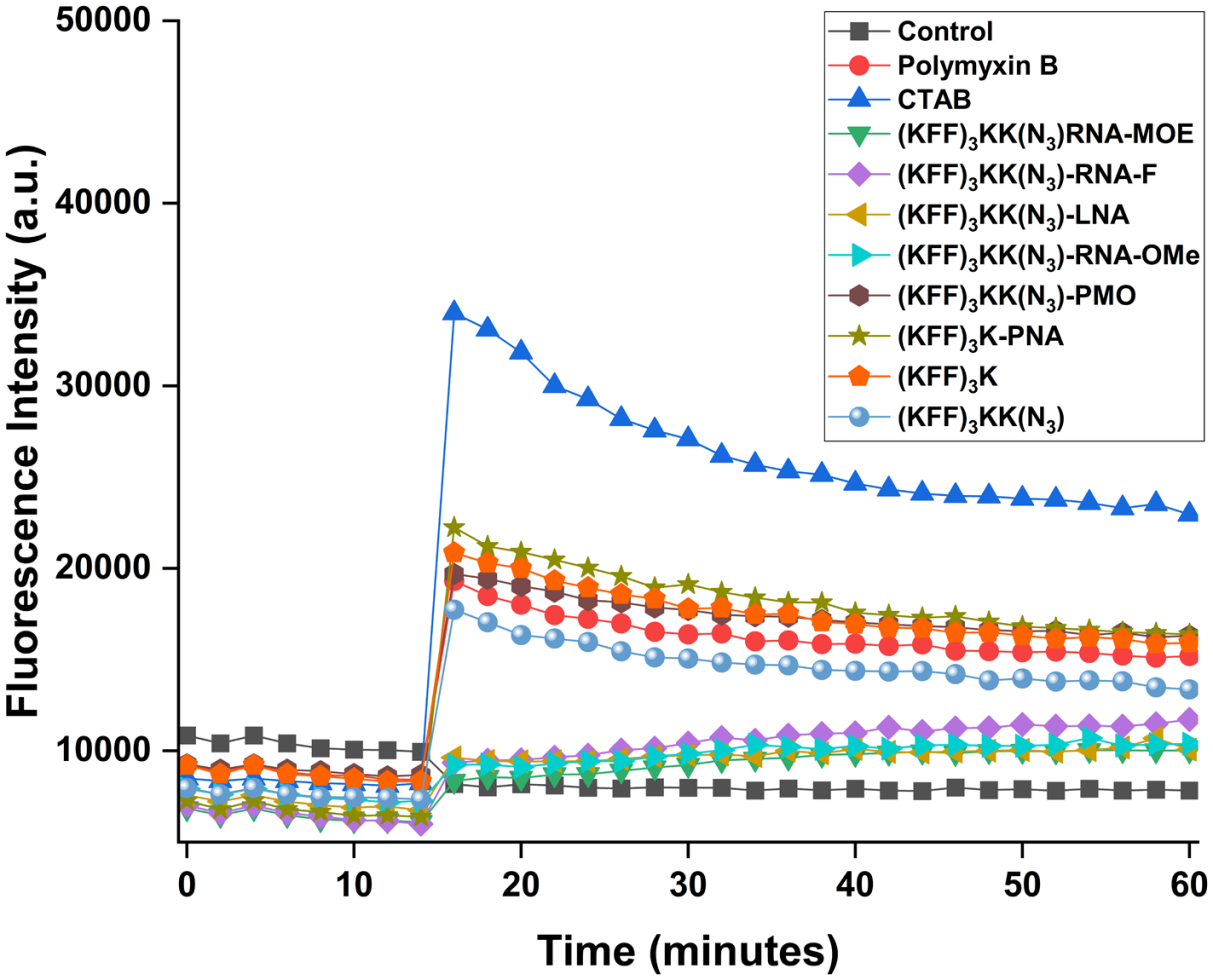
Outer membrane permeabilization of *Salmonella*. NPN fluorescence plotted over time. Different KFF-ASOs (10 µM) and controls were added after 14 min. CTAB (10 µM) and PMXB (0.4 µM) were used as positive controls, water as a negative control. A representative example of two independent experiments is shown.

Because the negASOs are ineffective in bacterial cells despite their activity in cell-free systems, we investigated if these ASOs affected the ability of the KFF carrier peptide to induce OM permeabilization. Therefore, we used the hydrophobic fluorophore *N*-phenylnaphthylamine (NPN), a dye that turns fluorescent after binding to hydrophobic regions within cell membranes. This only occurs in cells with a compromised OM (Ghosh et al. 2016; Ellis et al. 2019). CTAB (a surfactant) and Polymyxin B (PMXB), known permeabilizers of the bacterial OM, cause a rapid increase in fluorescence when added to *Salmonella* in this assay (Fig. 10). Similarly, both KFF control peptides permeabilize the OM, and this ability is retained upon conjugation to PNA and PMO. However, when conjugated to negASOs, the KFF carrier is unable to permeabilize the OM (Fig. 10). This could potentially be due to electrostatic interactions between the negASOs and the positively charged KFF. Overall, these results indicate that conjugation of the negASOs to the KFF peptide compromises its cell membrane permeabilizing activity.

### negASOs are not delivered efficiently inside *Salmonella* by the KFF peptide

Finally, to directly test if the ASO conjugates translocate into *Salmonella*, we used MALDI mass-spectrometry (MS) to identify ASO fragments in the bacterial periplasm after treatment with 10 µM KFF-ASO conjugates for 15 min (Table 2; Supplemental Figs. S7 and S8). Both PNA and PMO fragments were detected in the periplasmic fraction (Supplemental Fig. S7). The KFF-PNA fragments corresponded to either peptide-cleaved PNA or PNA attached to a single unit of KFF. This is in line with a study that reported that the KFF peptide is cleaved within the periplasm before translocation into the cytoplasm (Yavari et al. 2021). In the case of PMO, only the PMO fragment was observed in the periplasm, suggestive of KFF cleavage. Importantly, none of the negASOs were detected in this subcellular fraction (Supplemental Fig. S8), likely explaining the lack of antibacterial effects against *Salmonella*.

**TABLE 2.** Mass-spectrometry-based detection of antisense oligomer-peptide fragments in *Salmonella* periplasm and cytoplasm 15 min post-treatment

KFF-ASO	Periplasm	Fragments detected
(KFF)_3_K-PNA	✓	PNA (+KFF)
(KFF)_3_KK(N_3_)-PMO	✓	PMO
(KFF)_3_KK(N_3_)-LNA	ND	-
(KFF)_3_KK(N_3_)-RNA-F	ND	-
(KFF)_3_KK(N_3_) RNA-MOE	ND	-
(KFF)_3_KK(N_3_)RNA-OMe	ND	-

(ASO) Antisense oligomer, (PNA) peptide nucleic acid, (PMO) phosphorodiamidate morpholino, (LNA) 2′–4′-locked RNA, (RNA-F) 2′-fluorinated RNA, (RNA-MOE) 2′-methoxyethylated RNA, (RNA-OMe) 2′-methylated RNA, (ND) not detected.

## DISCUSSION

Despite their successful clinical application for treating genetic disorders in humans, ASOs are yet to be established as antibacterial drugs. For their effective clinical use, several important questions need to be answered. These include the identification of the best ASO modality, the best ASO carrier, the range of potential target genes and the overall bacterial response to ASO modalities. We have been trying to address these questions systematically with the ultimate goal of developing ASOs as programmable, species-specific antibiotics in complex bacterial communities (Vogel 2020; Popella et al. 2021, 2022). In the current study, we focused on a direct comparison of the antisense properties of seven different ASO modalities in the model pathogen *Salmonella*. Our results are provided as an overview in Table 3, and we will discuss our findings and directions for future studies below.

**TABLE 3.** Summary of the comparison between the different antisense oligomers

ASO modality	mRNA hybridization (EMSA)	Inhibition of translation in vitro	Antibacterial activity (MIC, µM)^a^	*acpP* depletion (RNA-seq)^a^	AcpP protein reduction	Outer membrane permeation^a^	Uptake inside bacteria (MS)^a^
RNA-F	++	++	>10	−	−	−	−
RNA-OMe	−	−	>10	−	−	−	−
RNA-MOE	++	+++	>10	−	−	−	−
LNA	+++	+++	>10	−	−	−	−
DNA-PTO	−	−	>10	−	−	−	−
PMO	+	++	10	+	+	++	+
PNA	+++	+++	1.25	++	++	++	+

(ASO) Antisense oligomer, (EMSA) electrophoretic mobility shift assays, (MIC) minimum inhibitory concentration, (MS) mass-spectrometry, (RNA-F) 2′-fluorinated RNA, (RNA-OMe) 2′-methylated RNA, (RNA-MOE) 2′-methoxyethylated RNA, (LNA) 2′–4′-locked RNA, (PTO) phosphorothioate, (PMO) phosphorodiamidate morpholino, (PNA) peptide nucleic acid.

^a^The ASO was conjugated to the carrier peptide. Effect strength is represented by additional (+) signs.

### ASO chemistry and binding affinity

Several ASO drugs that are based on diverse nucleotide modalities including DNA-PTO, RNA-MOE, RNA-OMe, RNA-F, and PMO have been approved for the treatment of human diseases (Bost et al. 2021; Crooke et al. 2021; Egli and Manoharan 2023; Hall 2023). In terms of pharmacokinetics, pharmacodynamics, and bioavailability in the human body, each modality has different properties (Herkt and Thum 2021). In addition, the RNA binding affinity of the ASO is also affected by these modifications. For example, RNAs containing 2′-modified sugars form more stable duplexes, whereas modifications on the phosphate backbone generally lower duplex stability (Freier and Altmann 1997). Here, we compared these different ASO modalities with PNA, currently the most commonly used modality in antimicrobial research. In general, our results are in agreement with the trends observed in structure–stability studies with different chemically modified nucleotide duplexes (Freier and Altmann 1997). Except for DNA-PTO, all ASOs were able to interact with their target transcript at high concentrations and to inhibit translation in vitro. PTO linkages are known to reduce affinity for complementary RNA (Freier and Altmann 1997), and it is likely that the DNA-PTO used in our experimental setting is not long enough to mediate efficient binding. Interestingly, LNA and RNA-MOE showed a stronger affinity to target mRNA compared to the neutral PNA and PMO. This makes LNA and RNA-MOE interesting alternatives to the neutral ASOs, as the phosphate groups of their backbone impart aqueous solubility to the ASO. Poor water solubility is a general drawback of PNAs.

It is also important to note that using four different types of in vitro measurements (EMSA, *T*_m_, MST, and in vitro translation) shows that it is advisable to use more than one method to predict productive target mRNA engagement when evaluating antibacterial ASO modalities, at least when the ASO moiety needs to be kept short.

To improve the binding properties of ASOs, there are two possible routes: increasing their length and increasing their affinity. Although the former might compromise their internalization, the use of alternative delivery vehicles might circumvent that problem. Alternatively, to improve affinity, ASOs can be further modified. For example, in the case of PNAs, the modification of the γ-position of the *N*-(2-aminoethyl) glycine unit reduces self-aggregation and improves solubility and duplex stability (Pradeep et al. 2023).

### The KFF peptide as delivery vehicle

For our side-by-side comparison of different ASO modalities, it was essential to identify a delivery vehicle that had been reported to deliver both neutral and negASOs. In addition, we needed a CPP that does not have a large effect on the global bacterial transcriptome, which otherwise would make the RNA-seq results difficult to interpret. KFF was the only choice in that regard (Geller et al. 2003; Meng et al. 2015; Popella et al. 2021). Although the KFF peptide served as a good starting point for our ASO comparison, it is important to highlight its shortcomings. First, the KFF peptide is susceptible to proteolytic cleavage, which hinders its systemic use. The use of noncanonical amino acids (such as the D-form or other nonnatural amino acids) and peptidomimetic approaches might circumvent this problem (Kalafatovic and Giralt 2017; Yavari et al. 2021). Second, it itself possesses antibacterial efficacy at around 30 µM (Macyszyn et al. 2023). Third, KFF is presumed to carry out cell penetration primarily by pore-formation or by other means of interaction with the bacterial membrane, thereby causing envelope stress (Yavari et al. 2021; Macyszyn et al. 2023). To a certain extent, this is reflected in our RNA-seq experiments, where genes related to CAMP resistance were affected by almost all KFF-ASO conjugates. This is not ideal as envelope stress affects several pathways in bacteria (as is evident in Figs. 6 and 7).

### The net charge of KFF-ASO conjugates affects antibacterial activity

The KFF peptide contains four units of the cationic amino acid lysine, and six units of the hydrophobic amino acid phenylalanine. Conjugation of KFF to the charge-neutral PNA or PMO retains the positive charge of the KFF peptide. In contrast, the negASO-KFF conjugates have a net negative charge. This might hinder their interaction with the bacterial membrane, which also has an overall negative charge (Epand and Epand 2009). In addition, electrostatic interactions between the negASO and the KFF peptide can lead to intra- and intermolecular aggregates. This might be the reason why the conjugation of CPPs to charged-backbone ASOs has not been widely explored, although there are a few successful examples of such conjugates (Meng et al. 2015). Our observation that KFF-conjugated negASOs are unable to cross the bacterial OM of *Salmonella* (within 15 min post-treatment) indicates that these ASOs might have a negative effect on the membrane-permeating properties of KFF. Although some of the negASOs cause growth retardation of *Salmonella* in our experimental settings, this effect cannot be explained by specific reduction of its cellular target on RNA or protein level. Our RNA-seq data show that with the exception of RNA-MOE, negASOs trigger the activation of stress response pathways related to PhoP/Q, PMR A/B, and CAMP, indicating that these negASOs do interact with the cell membrane and might lead to stress-induced toxicity.

### negASOs require alternative delivery vehicles

Our data show that LNA and RNA-MOE are potent translational inhibitors of the *acpP* target transcript in vitro. In fact, they outperform PNA and PMO in this assay. However, they are not efficiently delivered across the bacterial membrane by the KFF carrier peptide. This is in contrast to an earlier study that showed that a KFF-delivered LNA-based ASO is active against *S. aureus* both in vitro and in a murine model of infection (Meng et al. 2015). Because *S. aureus* is a Gram-positive bacterium, delivery will likely have distinct requirements compared to Gram-negative *Salmonella*. Nonetheless, the replacement of the KFF peptide with peptides that bear more cationic amino acids might be more effective in delivering negASOs. Alternatively, the negative charges of the phosphate backbone can also be used to complex negASOs with other cationic carriers such as bolaamphiphiles (Sharma et al. 2018). This will mitigate the net negative charge of the complex and may lead to better delivery. Because bacteria lack eukaryotic uptake mechanisms that allow the uptake of nanoparticles, new approaches need to be developed. There have been reports of successful delivery of ASOs across the bacterial membrane with vitamin B_12_, DNA nanocages, bolaamphiphiles, siderophores, and other macromolecules (Hegarty et al. 2016; Zhang et al. 2018; Równicki et al. 2019; Bost et al. 2021; Long et al. 2021; Pereira et al. 2021; Liu et al. 2023; Pals et al. 2024). These recent advances in the design of macromolecular carriers might open up more avenues for the use of negASOs as antibacterials.

### PNA versus PMO

Although PNAs and PMOs have been compared before with respect to synthesis, stability, backbone flexibility, aqueous solubility, sequence specificity, and target binding (Summerton 2003), there has not been a direct comparison of their antibacterial activity. In general, PNAs have a higher binding affinity to RNA, whereas PMOs are more soluble in water (Summerton 2003). Here, we find that PNA is more effective than PMO in inhibiting the translation of *acpP* both in vitro and within *Salmonella*. Of note, the molecular mass of the PMO conjugate is ∼1.4 kDa higher than that of the PNA conjugate. Whether this has any influence on bacterial internalization is unclear.

Another point of consideration is the effect of the linker that was used to conjugate PMO to the KFF peptide in this study. PNA-KFF conjugates can be synthesized as fusion peptides due to the PNA pseudo-peptide backbone. In contrast, the PMO conjugate was linked by copper-free click chemistry in our study. It is, therefore, structurally different from the peptide-PMO conjugates reported in the literature where the peptide is conjugated to the PMO using amide-coupling chemistry (Tilley et al. 2006; Mellbye et al. 2009). Although there is no direct comparison of the activity between click-coupled and amide-coupled peptide-PMO conjugates, we do not observe a large difference in the antibacterial activity of click-coupled PMO compared to an earlier study using amide-coupled PMO (Tilley et al. 2006). Thus, the linker is unlikely to strongly affect the antimicrobial activity of PMO.

Interestingly, the transcriptomic response of *Salmonella* to PMO is different compared to PNA. Whereas PNA has little effect on the global transcriptome when coupled to the KFF peptide, PMO activates membrane stress responses, similar to the unconjugated peptide controls. This is surprising, because both have a neutral backbone and are coupled to the same peptide carrier. The reason for this observation remains unknown. We have recently shown that 9mers PNAs can retain full activity (Popella et al. 2022), and therefore it would be interesting to see if using shorter PMO derivatives affects the extent of the membrane stress response. Regardless, this effect needs to be taken into account when using PMO in applications other than pathogen killing, for example, precision modulation of gene activity in genetically intractable microbes.

We observe that PNA has a stronger effect than PMO on the transcript levels of *acpP*, which is also reflected on the protein level. Therefore, in our experimental setting using *acpP* as a target and treating bacteria in culture, PNA outperforms PMO as an antisense antibacterial. However, it should be stressed that PMO has shown great efficacy in animal experiments (Geller et al. 2018; Moustafa et al. 2021), which warrants a more systematic comparison of PNA versus PMO with respect to length and a larger number of targets in the future.

### Limitations of the study

Here, we have compared the antibacterial potential of different ASOs in vitro and in bacterial cell culture, using a single target mRNA and a fixed number of bases, thus limiting conclusion with respect to other targets and ASO sequence lengths. Our study also does not address the pharmacodynamics and pharmacokinetics of the peptide–ASO conjugates in animal models. To compare the effectiveness of the different ASO modalities in animal models of infection, it is essential to develop alternative delivery strategies, especially for the effective intrabacterial translocation of negASOs such as RNA-MOE and LNA.

## MATERIALS AND METHODS

### Oligomers, reagents, and bacterial strains

The antisense oligonucleotides bearing phosphate/PTO backbones were purchased from Biomers.net GmbH. PMOs were purchased from GeneTools, LLC. PNAs, peptides, and peptide-conjugated PNAs (PPNAs) were obtained from Peps4LS GmbH. The quality and purity of these constructs were verified by MS and HPLC by the respective companies. ASOs, peptides, and PPNAs (Table 1) were dissolved in water and heated at 55°C for 5 min before determining the concentration by using a NanoDrop spectrophotometer (*A*_260 nm_ for ASO and KFF-ASO). The peptides were weighed out and dissolved in water to reach a concentration of 10 mg/mL. Aliquots of ASOs and peptides were stored at −20°C and heated at 55°C for 5 min before preparing working dilutions. Low-retention pipette tips and low-binding Eppendorf tubes (Sarstedt) were used throughout. *Salmonella enterica* serovar Typhimurium strain SL1344 wild type (WT) (provided by D. Bumann, Biocenter Basel, Switzerland; internal strain number JVS-1574) and SL1344 AcpP::3xFLAG (chromosomal fusion of full-length *acpP* to 3xFLAG-tag as described earlier [Datsenko and Wanner 2000]; internal strain number JVS-12398) were used in this study. Bacteria were cultured in noncation adjusted Mueller-Hinton Broth (MHB, BD Difco, Thermo Fisher Scientific) with aeration at 37°C and 220 rpm shaking.

### Electrophoretic mobility shift assay

The EMSA was performed with a Cy5 5′-labeled RNA (purchased from Eurofins) encompassing the sequence spanning nucleotides −20 to +20 of *acpP* relative to the translation start codon. For annealing, TM buffer (10 mM Tris-HCl, 50 mM MgCl_2_) was used. First, 20 µM RNA was denatured by incubation at 95°C for 5 min followed by placement on ice. Then 1 µL of RNA (spiked with or without 1 µL of yeast tRNA [Ambion at a concentration of 10 mg/mL]) was added to PCR tubes containing 8 µL of water. To this, 1 µL of ASOs at the indicated concentrations (6.25–50 µM) was added to adjust ASO:RNA ratios of 0.3:1 to 2.5:1. As negative control, water was added instead of ASO, while a PNAscr sequence served as sequence-unspecific negative control (Fig. 2; Supplemental Fig. S2). These mixtures were incubated for 30 min at room temperature (RT), followed by the addition of 10 µL 2× gel loading (GL) buffer for subsequent loading on native 15% PAA RNA gel without urea (20 µL/sample; 6 µL of a 50 bp DNA marker + 14 µL 1× GL). The gel was run at 100–120 V for 2–3 h, concomitantly stained with SYBR gold (Thermo Fisher Scientific) for 15 min and imaged on Geldoc (Isogen Life Science B.V.). Figure 2 shows a representative image out of two replicates.

### Thermal UV melting curves

Three hundred microliters of 10 nt long target mRNA (2 µM) was mixed with 300 µL of each of the ASOs (2 µM) in phosphate buffer (100 mM NaCl, 10 mM phosphate, pH 7.0). Thermal UV melting curves were recorded at Agilent Cary UV–Vis Multicell Peltier. Before measurement, the samples were overlaid with silicon oil to prevent evaporation during heating. Five temperature ramps with a heating/cooling rate of 0.5°C/min between 10°C and 90°C at 260 nm were collected. All the melting curves were plotted using OriginPro 2023, fit to a two-state transition model with upper and lower baselines to determine *T*_m_ as previously reported (Dietzsch et al. 2022).

### Determination of binding affinities using microscale thermophoresis

For each binding experiment, Cy5 5′-labeled RNA (as described above) was diluted to 10 nM in buffer A (50 mM Tris-HCl pH 7.6, 250 mM KCl, 5 mM MgCl_2_, 1 mM DTT, 5% glycerol supplemented with 0.05% Tween 20). A series of 16 tubes with different ASO dilutions (500–0.015 nM) were prepared in buffer A. For measurements, each ASO dilution was mixed with one volume of labeled RNA, which led to a final concentration of 5 nM labeled RNA and 250–0.007 nM of ASOs. The reaction was mixed by pipetting and incubated for 30 min at RT in the dark. Capillary forces were used to load the samples into Monolith NT.115 Premium Capillaries; measurements were performed using a Monolith Pico instrument (NanoTemper Technologies Gmbh) at an ambient temperature of 25°C. Instrument parameters were adjusted to 5% LED power, medium MST power, and MST on-time of 2.5 s. An initial fluorescence scan was performed across the capillaries to determine sample quality, which was followed by 16 thermophoresis measurements. Measurements that showed fluorescence inhomogeneity or aggregation were excluded. Data of two independent measurements were analyzed for the ΔFnorm value determined by the MO Affinity Analysis software (NanoTemper Technologies Gmbh). The fitting for EC_50_ values was done using the Hill equation within the MO Affinity Analysis software. Data are presented as the mean EC_50_ values of two independent experiments.

### Synthesis of target RNA using in vitro transcription

For the generation of target RNA::*gfp* fusion constructs, we used a previously established protocol (Popella et al. 2022) and the oligonucleotides JVO-19760/JVO-19761 (Supplemental Table S1) to amplify the genomic region spanning nucleotides –40 to +51 relative to the translational start codon of *acpP*. The sense oligonucleotide was fused to a T7 promoter sequence for subsequent transcription of the final fusion construct in vitro. The antisense oligonucleotide contained a 30 nt overlapping region with *gfp* for subsequent fusion PCR with full-length *gfp* (pXG-10; JVO-19762/JVO-19763) (Supplemental Table S1). The PCR products were purified with NucleoSpin Gel and PCR Clean-up (Macherey-Nagel) according to the manufacturer's instructions, and DNA concentration was quantified with a NanoDrop spectrophotometer (*A*_260 nm_).

PCR products were used for T7 RNA polymerase-driven transcription in vitro using the MEGAscript T7 kit (Ambion/Thermo Scientific), according to the manufacturer's instructions and analogous to our previous publication (Popella et al. 2022). The concentration of the RNA solution was quantified with Qubit (Fisher Scientific), and the expected product size and RNA integrity were verified on a 6% PAA, 7 M urea gel.

### In vitro translation and western blotting

For in vitro translation of target RNA::*gfp* fusion constructs, the PURExpress In Vitro Protein Synthesis Kit (New England Biolabs, E6800L) was used as described previously (Popella et al. 2022). Briefly, the final volume of each in vitro translation reaction was set to 10 µL. One microliter of different concentrations of ASOs ranging from 10 to 0.625 µM was added to 1 µL of in vitro transcribed heat-denatured RNA (1 µM) to adjust the final RNA concentration to 100 nM. The mixtures were preincubated at 37°C for 5 min. Then 4 µL of Solution A and 3 µL of Solution B were added to the preannealed mixtures. After 2 h at 37°C, protein samples were denatured in 1× reducing protein loading buffer (62.6 mM Tris–HCl pH 6.8, 2% SDS, 0.1 mg/mL bromphenol blue, 15.4 mg/mL DTT, 10% glycerol) at 95°C for 5 min.

To visualize in vitro translated proteins, samples were separated on SDS-PAA (12% PAA) gels, with subsequent semi-dry western blot transfer on nitrocellulose membranes. Membranes were blocked with 10% skim milk (in 1× TBS-T) for 1 h and probed with anti-GFP antibody (1:1000; Sigma-Aldrich) in 5% skim milk (in 1× TBS-T) overnight at 4°C. After washing in 1× TBS-T and incubation with an HRP-conjugated secondary antibody (1:5000; Thermo Scientific) in 1× TBS-T, the membrane was incubated with a developing solution kit (Amersham ECL Select Western Blotting Detection Reagent), and protein levels were detected using an ImageQuant LAS 500 (GE Healthcare Life Sciences). Images were processed and band intensities quantified using ImageJ. Protein bands were normalized to the water control.

### Synthesis of ASO-peptide conjugates

Due to their pseudo-peptide backbone, KFF-PNA conjugates can readily be synthesized as a fusion peptide using a peptide synthesizer. The HPLC-purified KFF-PNA was purchased directly from Peps4LS GmbH. All other ASOs are synthesized on an oligonucleotide synthesizer with an appended 5′ terminal dibenzocyclooctyne (DBCO) moiety, which enabled strain-promoted alkyne–azide cycloadditions (SPAAC) (copper-free click chemistry) with azides (Gordon et al. 2012). To facilitate coupling to KFF, a terminal lysine whose ε-amine was replaced by an azide was added as the carboxy terminal amino acid [sequence: (KFF)_3_KK(N_3_)].

All ASOs with a DBCO terminated linker (Fig. 1B) were purchased from Biomers with the exception of PMO, which was purchased from GeneTools, LLC. The DBCO terminated linker was not available from GeneTools, LLC, so we used PMO terminating with a cyclooctyne linker. Of note, the antisense PMO-peptide conjugates previously reported to have antibacterial activity did not use click chemistry for conjugation to the peptides but use amide-coupling chemistry (Deere et al. 2005; Wesolowski et al. 2013).

In a typical reaction, one equivalent of ASO (167 nmol in 50 µL water) with the DBCO moiety was added to three equivalents of (KFF)_3_KK(N_3_) (501 nmol in 75 µL water). The reaction was performed at 4°C overnight. Precipitation was observed for some of the compounds (e.g., LNA, DNA-PTO, RNA-MOE) when mixed with the (KFF)_3_KK(N_3_) peptide, which was usually resolved by adding equal volumes of acetonitrile or formamide or by heating it slightly. The ASO-CPP conjugate was separated from the reactants by running them on 20% polyacrylamide gel, excising the appropriate bands, and extracting them into TEN buffer (0.1 M Tris-Cl pH 8.0, 0.01 M EDTA pH 8.0, 1 M NaCl). The compounds were then purified using size exclusion chromatography (Äkta pure, Cytiva). The PMO conjugate was purified by HPLC (Jasco). Each ASO-CPP conjugate was characterized using MALDI MS (Supplemental Fig. S5). The HPLC traces of PMO and PNA conjugates have been provided in Supplemental Figure S5 as well. The samples were stored as lyophilized powders at −20°C for further use.

### *Salmonella* growth assays

An overnight bacterial cell culture was diluted 1:100 in fresh MHB and grown to OD_600_ 0.5. The culture was then diluted to ∼10^5^ cfu/mL in noncation-adjusted MHB. Subsequently, 190 µL bacterial solution was dispensed into a 96-well plate (Thermo Fisher Scientific) along with 10 µL of a 200 µM solution of the ASOs. Growth was monitored by measuring the OD at 600 nm every 20 min in a Synergy H1 plate reader (Biotek) with continuous double-orbital shaking (237 cpm) at 37°C for 24 h.

### RNA isolation for sequencing

RNA isolation was performed using previously published protocols (Popella et al. 2021, 2022). Bacterial overnight cultures were diluted 1:100 in fresh MHB and grown to OD_600_ 0.5. The cultures were diluted to ∼10^6^ cfu/mL in noncation-adjusted MHB. Afterward, an aliquot of the bacterial solution was transferred into 5 mL low-binding tubes (LABsolute) containing the ASOs solution (adjusted such that the final concentration was 5 μM for all tested compounds). As a negative control, cells were treated with the respective volume of sterile nuclease-free water, which was used as solvent for the ASOs. After incubating the samples for 15 min at 37°C, RNAprotect Bacteria (Qiagen) was added according to the manufacturer's instructions. Following a 10-min incubation, cells were pelleted at 4°C and 21,100*g* for 20 min. The supernatant was discarded and pellets were either directly used or stored at −20°C (<1 d) for subsequent bacterial RNA isolation.

Total RNA was purified from bacterial pellets using the miRNeasy Mini kit (Qiagen) according to protocol #3 described in Popella et al. (2021). Briefly, cells were resuspended in 0.5 mg/mL lysozyme (Roth) in TE buffer (pH 8.0) and incubated for 5 min. Afterward, RLT buffer supplemented with β-mercaptoethanol, and ethanol were added according to the manufacturer's instructions. After sample loading, column wash-steps were performed according to the manual. RNA concentration was measured with a NanoDrop spectrophotometer.

### RNA-sequencing

For transcriptomic analyses, RNA samples were processed and subjected to RNA-seq at the Core Unit SysMed (University of Würzburg). Sequencing was run using a previously published protocol (Popella et al. 2022). Briefly, RNA quality was checked using a 2100 Bioanalyzer with the RNA 6000 Pico/Nano kit (Agilent Technologies). RNA samples were DNase-treated using the DNase I kit (Thermo Fisher), followed by ribosomal RNA depletion using Lexogen's RiboCop META rRNA Depletion kit protocol according to the manufacturer's recommendation. Subsequently, cDNA libraries suitable for sequencing were prepared using CORALL Total RNA-Seq Library Prep protocol (Lexogen) according to the manufacturer's recommendation with 14–25 PCR cycles. Library quality was checked using a 2100 Bioanalyzer with the DNA High Sensitivity kit (Agilent Technologies). Sequencing of pooled libraries, spiked with 5% PhiX control library, was performed at 10 million reads/sample in single-end mode with 75 nt read length on the NextSeq 500 platform (Illumina) using High output sequencing kits. Demultiplexed FASTQ files were generated with bcl2fastq2 v2.20.0.422 (Illumina).

### RNA-seq data analysis

RNA-seq data analysis was performed similarly to our previous analysis of PNA activity in *S. enterica* (Popella et al. 2021). Briefly, adapters and low-quality bases (Phred quality score <10) of raw reads were trimmed using BBduk. Then the RNA-seq reads were mapped to the *S. enterica* serovar Typhimurium reference genome and the three plasmids pSLT SL1344, pCol1B9 SL1344, and pRSF1010 SL1344 (Kröger et al. 2012) using BBMap (v38.18), and genomic features were assigned using the featureCounts command of the Subread (2.0.1) package (Liao et al. 2014). For the coverage plots in Figure 8, the bamCoverage (v3.3.2) method of the deepTools platform was applied (Ramírez et al. 2014).

### Normalization and differential expression analysis

RNA-seq analysis was performed using packages from the R/Bioconductor project. Raw read counts of all conditions were imported into edgeR (v3.34.1) (Robinson and Oshlack 2010) and analyzed for differential expression. To filter low-count reads, features with <1.38 CPM in at least five libraries were ignored for further analysis. The cutoff of 1.38 CPM was set to 10/*L*, with *L* being the minimum library size across all samples in millions, as described in Chen et al. (2016a). Libraries were normalized using edgeR's trimmed mean of *M* (TMM) values normalization (Robinson and Oshlack 2010). The glmFit function was applied to estimate quasi-likelihood dispersions, and contrasts for differential expression analysis were tested using the glmQLTest function. Features with an absolute FC > 2 and FDR (Benjamini and Hochberg 1995) adjusted *P*-values < 0.001 were considered significantly differentially expressed. The results were plotted as heatmaps using the ComplexHeatmap (2.12.0) package (Gu 2022).

### KEGG pathway analysis

To perform gene set analysis, features were assigned to KEGG pathways (Kanehisa et al. 2023) with the R package KEGGREST (v1.32.0). Additionally, genes were assigned to gene sets of regulons curated in Westermann et al. (2016). To assess enriched KEGG pathways and regulons, rotation gene set testing was performed using the FRY function of the ROAST package (Wu et al. 2010). Top up- or down-regulated gene sets covering >10 genes and an FDR-adjusted *P*-value < 0.01 (marked with an asterisk) in at least one condition were visualized in Figure 7. The color in the heatmap denotes the median log_2_FC of the respective gene set. The figure shows gene sets ranking among the top 10 significant gene sets (lowest *P*-value) in at least one sample.

### Quantification of intracellular AcpP protein levels post-ASO treatment

*Salmonella* WT and SL1344 AcpP::3xFLAG were grown overnight in noncation adjusted MHB. Bacterial cultures were diluted 1:100, grown to OD_600_ 0.5, and subsequently diluted to ∼10^6^ cfu/mL in MHB. Then, 1.9 mL of each diluted bacterial solution was transferred into 5 mL low-binding tubes, preincubated at 37°C for 10 min, and treated with 100 µL of ASOs to adjust a final concentration of 5 µM. Water was added as negative control. The treatment with PNA and PMO was performed with aeration at 37°C and 220 rpm shaking for 30, 60, and 120 while for all other ASOs, they were treated for 120 min only. Subsequently, the cells were collected by centrifugation at 4°C and 16,000*g* for 5 min, resuspended in 1× Protein Loading Buffer (62.6 mM Tris–HCl pH 6.8, 2% SDS, 0.1 mg/mL bromophenol blue, 15.4 mg/mL DTT, 10% glycerol) and denatured at 95°C for 10 min.

For detection of AcpP::3xFLAG protein levels, samples were separated on a 12% SDS-PAA gel and transferred onto a nitrocellulose membrane via semi-dry western blotting. The membrane was probed with an α-FLAG antibody (Sigma; 1:1000 in 1× TBS-T containing 3% BSA) at 4°C overnight. After washing in 1× TBS-T, the membrane was incubated with a secondary HRP-conjugated antimouse antibody (Thermo Scientific; 1:5000 in 1× TBS-T containing 1% BSA) at RT for 1 h. Excessive antibody was removed by repeated wash-steps in 1× TBS-T, and the membrane was developed by an Amersham ECL Select Western Blotting Detection Reagent Kit (Cytiva).

### Outer membrane permeabilization

The experiments were performed by modifying previously published protocols (Ghosh et al. 2016; Ellis et al. 2019; Zheng et al. 2021). *Salmonella* (WT SL1344, JVS-1574) were grown overnight in 4 mL MHB medium at 37°C (with shaking at 220 rpm). Then the culture was diluted 1:100 and grown until OD_600_ 0.5 (∼2 × 10^8^ cfu/mL). This culture was harvested and washed two times with 5 mM HEPES and further diluted to OD_600_ 0.2 in 5 mM HEPES supplemented with 5 mM Glucose (pH 7.2). These cells were then incubated with NPN (Sigma) at a final concentration of 10 µM. One hundred and ninety microliters of NPN-treated cells were transferred into 96-well back plates with transparent bottoms and fluorescence (excitation = 350 nm, emission = 420 nm) was recorded for 60 min (reading was performed every minute) using a Synergy H1 plate reader (Biotek). On the 14th minute, 10 µL of the test compounds (10 µM final concentration) was added into the wells. CTAB (Sigma) 10 µM (final concentration) or PMXB (Sigma) (final concentration of 0.4 µM) were used as positive controls, while water was used as negative control. The fluorescence of the wells was monitored using the same settings as above. The plots were created using OrginPro 2023. The experiment was performed twice and Figure 10 is a representative example of two independent experiments.

### MALDI-TOF-based analysis of ASO-peptide conjugate uptake into bacterial cells

The experiments were performed by modifying previously published protocols (Yavari et al. 2021). From an overnight culture of *Salmonella*, a second culture was inoculated (1:100) and grown to OD_600_ of 0.5 (corresponding to ∼2 × 10^8^ cfu/mL). These bacterial cells were washed twice in MHB and resuspended in 95 µL of MHB with an additional incubation at 37°C for 5–10 min. Sterile ASO-peptide conjugates were prepared at concentrations of 200 µM. Five microliters of the 200 µM working solutions were added to 95 µL of bacteria in culture media, leading to a final concentration of the compounds of 10 µM. Five microliters of H_2_O was added as a negative control. The tubes were incubated at 37°C for 15 min with shaking at 300 rpm. Subsequently, they were centrifuged at 8000*g* for 5 min at 4°C. The supernatant was aspirated, and the pellet was washed with 0.01 M Tris-HCl before being resuspended in 200 µL of 0.01M Tris-HCl containing 0.5 M Sucrose, 150 µg/mL lysozyme, 10 mM EDTA and incubated at 37°C overnight. These tubes were then centrifuged at 2000*g* at 4°C for 10 min. The supernatant obtained represents the periplasm. The periplasm fragment was then mixed with 2,6-Dihydroxyacetophenone (DHAP) in ACN 1:1, spotted on a MALDI plate, and analyzed for MALDI-TOF-MS in a Bruker DaltonicsUltrafleXtreme Instrument. The experiment was performed two times, and data of one representative experiment are shown.

## DATA DEPOSITION

Our RNA-seq data set has been deposited with NCBI Gene Expression Omnibus (GEO) under accession number GSE232819 (https://www.ncbi.nlm.nih.gov/geo/query/acc.cgi?acc=GSE232819). The code used for data analysis is available at GitHub (https://github.com/BarquistLab/aso_screen_ghosh_et_al_2023).

## SUPPLEMENTAL MATERIAL

Supplemental material is available for this article.

## Figures and Tables

**Chandradhish Ghosh:**
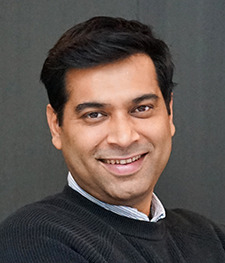


## MEET THE FIRST AUTHOR

***Meet the First Author(s)* is an editorial feature within *RNA*, in which the first author(s) of research-based papers in each issue have the opportunity to introduce themselves and their work to readers of *RNA* and the RNA research community. Chandradhish Ghosh is the first author of this paper, “A comparative analysis of peptide-delivered antisense antibiotics using diverse nucleotide mimics.” Chandradhish is a senior postdoctoral researcher at Helmholtz Institute for RNA-based Infection Research, and his current research is focused toward developing antisense oligonucleotides as species-specific antimicrobials.**

**What are the major results described in your paper and how do they impact this branch of the field?**

Antisense antibiotics have the unique advantage that they can be programmed to target a single pathogen in the presence of other communities of bacteria. But to get to that stage, we need to solve many problems. One of them is what is the best antisense modality? Toward that we have now systematically compared all of the successful modalities that are commonly used in oligonucleotide-based drugs. We show that using cell-penetrating peptides as delivery agents (current gold standard), ASOs with peptide nucleic acid (PNA) backbones work the best. However, if we find a compatible delivery agent, ASOs with backbones made of locked nucleic acids (LNA) or 2′-methoxyethyl RNA (RNA-2′MOE) might just be better than PNA. This systematic comparison of different ASO modalities will help future designs of ASO antibiotics—especially toward finding a better ASO carrier.

**What led you to study RNA or this aspect of RNA science**?

During my PhD, I was introduced to the problem of antimicrobial resistance and the need to discover alternatives to currently used antibiotics. Antisense technology represents a promising antibiotic alternative and offers advantages over conventional ones. It can be programmed to be species-specific, targeting only pathogenic bacteria. Resistance due to mRNA mutations can be reversed by changing the ASO sequence. Additionally, they can target any transcript, potentially reinstating the sensitivity of drug-resistant bacteria to existing antibiotics. Hence, I started working on this really exciting area of research.

**During the course of these experiments, were there any surprising results or particular difficulties that altered your thinking and subsequent focus?**

I found it intriguing that the cell-penetrating peptide (KFF) we utilized in our experiments failed to transport any negatively charged ASOs (such as LNA or RNA-MOE) into the bacteria under our tested conditions. I anticipated some level of efficacy. This finding has prompted me to consider the potential for an alternative delivery method. If we can identify a vehicle capable of delivering these stronger binding ASOs, it could lead to a significant breakthrough in the field. Currently, this is the focus of my research, and I'm enthusiastic about the exciting prospects ahead.

**What are some of the landmark moments that provoked your interest in science or your development as a scientist?**

Since childhood, I've harbored a strong desire to become a scientist, influenced in part by a late cousin who was also a scientist. A pivotal moment during middle school occurred when I stumbled upon a caterpillar pupa and meticulously observed its transformation into a butterfly. This captivating experience really influenced me to take up natural sciences. As I delved into organic chemistry, I fell in love with it and knew that I wanted to design therapeutics. From that point onward, I've dedicated myself to this pursuit.

**If you were able to give one piece of advice to your younger self, what would that be?**

If I could offer one piece of advice to my younger self, it would be to strategize my career as a scientist. It's crucial to determine the type of science you're passionate about—whether it's basic or translational—and seek out organizations such as universities, research institutes, or companies that share your vision and values.

**Are there specific individuals or groups who have influenced your philosophy or approach to science?**

Indeed at every step of my career, my supervisors have influenced the way I look at science. During my PhD, my supervisor Professor Jayanta Haldar instilled a sense of courage in me that allows me to tackle problems that I do not have a background in. During my first postdoc with Professor Peter Seeberger, I learned how to think independently and to translate science beyond the laboratory or academia. My current mentor Professor Jörg Vogel has influenced me to think beyond the present and envision what science in the future might look like. Apart from my mentors, I draw inspiration from the versatility of research problems addressed by Professors George Whitesides, C.N.R. Rao, and Robert Langer.

**What are your subsequent near- or long-term career plans?**

I aspire to establish my own independent laboratory in the near future. However, it's not enough for me to conduct research solely within the confines of the laboratory; I'm also committed to translating my findings to benefit society. Whether in academia, industry, or a start-up, I am eager to lead efforts in designing new therapeutics and exploring innovative technologies. As long as I have the opportunity to drive progress in these areas, I will be fulfilled.
